# SARS-CoV-2 vaccination and infection elicit cross-neutralizing responses against clade 3 and 4 sarbecoviruses

**DOI:** 10.1038/s41467-026-71662-y

**Published:** 2026-04-16

**Authors:** Stephen D. Schmidt, Rahul Subramanian, Manjula Basappa, Robin Carroll, Megi Rexhepaj, Christine M. Posavad, Jeffrey A. Liao, Jennifer A. Bohl, Ananda Chowdhury, Alicen B. Spaulding, Bob C. Lin, Mike Castro, Richard A. Koup, Leonid A. Serebryannyy, Daniel C. Douek

**Affiliations:** 1 Vaccine Research Center, National Institute of Allergy and Infectious Diseases, National Institutes of Health, Bethesda, MD USA; 2 Infectious Diseases Clinical Research Consortium (IDCRC) Laboratory Operations Unit, Vaccine and Infectious Disease Division, Fred Hutchinson Cancer Center, University of Washington, Seattle, WA USA; 3 Department of Laboratory Medicine and Pathology, University of Washington, Seattle, WA USA

**Keywords:** SARS virus, Viral infection, Vaccines

## Abstract

Two sarbecoviruses, SARS-CoV-1 and SARS-CoV-2 that engage ACE2 through their receptor-binding domains, have caused major human outbreaks. The pandemic potential of sarbecoviruses has prompted the discovery and classification of bat and other zoonotic sarbecoviruses that are also able to use human ACE2 or ACE2 ortholog receptors for infection. However, the current human immunological landscape reactive to these SARS-CoV-2-related viruses is not well profiled. Using a panel of pseudotyped lentiviruses expressing only spike proteins, we assess serum neutralization activity against clade 3 and 4 (also designated as clade 1c) receptor binding domain classified sarbecoviruses in a cohort who received a primary series of COVID-19 mRNA vaccines as well as individuals before and after infection with BA.5 or XBB.1.5 variants. Detectable neutralizing responses against clade 3 and 4 sarbecoviruses are observed in both vaccinees and convalescents and are comparable in magnitude to titers against SARS-CoV-2 variants. Infection with XBB.1.5 increases neutralization titers against SARS-CoV-2 variants as well as against clade 3 and 4 sarbecoviruses. Collectively, our findings suggest that the current immunologic landscape of vaccination and infection may confer some level of immunity against a variety of clade 3 and 4 sarbecoviruses, which should inform future pandemic response and pan-sarbecovirus countermeasure efforts.

## Abstract (web summary)

Here, the authors show that primary SARS-CoV-2 vaccination series elicits cross-neutralizing responses against clade 3 and 4 (1c) sarbecoviruses. Continued SARS-CoV-2 variant exposures have matured responses to be both cross-reactive and potent.

## Introduction

Two major sarbecovirus outbreaks have occurred in the past two decades: SARS-CoV-1 in the early 2000s, with over 8000 reported cases and a case fatality rate of approximately 10%, as well as SARS-CoV-2 that emerged in 2019 and has resulted in millions of deaths worldwide^1,2^. Both of these sarbecoviruses use human angiotensin-converting enzyme 2 (ACE2) as a receptor for entry through contact with the receptor binding domain (RBD) of the viral spike protein^2^. Sarbecoviruses recently discovered in bats and other animals that are phylogenetically related to SARS-CoV-1 and -2, and have been shown to use human ACE2 or ACE2 orthologs, may pose a continuous zoonotic threat^3,4^. However, it is unclear if entry from phylogenetically similar sarbecoviruses would pose a greater threat due to an increased likelihood of zoonotic spillover or a lesser threat due to the chance of existing population-wide immunity.

Classification of sarbecoviruses using homology across the RBDs has defined four broad phylogenetic clades^3,5^. Clade 1 is the most characterized, as it contains SARS-CoV-1 (1a) and SARS-CoV-2 (1b) related viruses. Clades 3 and 4 (also designated as clade 1c) have been identified in bats, are suggested to bind and use ACE2 orthologs for entry^3^ and may therefore have broad tropism^6^, but are less studied. Therefore, we sought to assess immunity against clade 3 and 4 sarbecoviruses using viral neutralization titers, a correlate of SARS-CoV-2 protection^7–10^. We used a replication-incompetent pseudotyped lentivirus system containing a luciferase reporter gene and solely the spike proteins from previously described clade 3 and 4 sarbecoviruses. As our interest was in human immunity against viral infection using human ACE2 as a receptor, we used human ACE2 stably overexpressing 293 T cells as a model for cell entry. For clade 3 and 4 spike proteins that showed little to no cell entry, mutations in the RBD were introduced using a strategy described by Starr et al.^3^ (Supplementary Table 1). As references, we also produced replication-incompetent pseudotyped versions of SARS-CoV-2 variants of concern, a RaTG13 mutant, SARS-CoV-1, SARS-CoV-1-like SHC014, and NeoCov, a relative of the Middle East respiratory syndrome (MERS) coronavirus, which can efficiently enter cells overexpressing human ACE2 when a single point mutation is introduced^11^.

## Results

### Neutralization activity after 2-dose ancestral strain vaccination

**Fig. 1:**
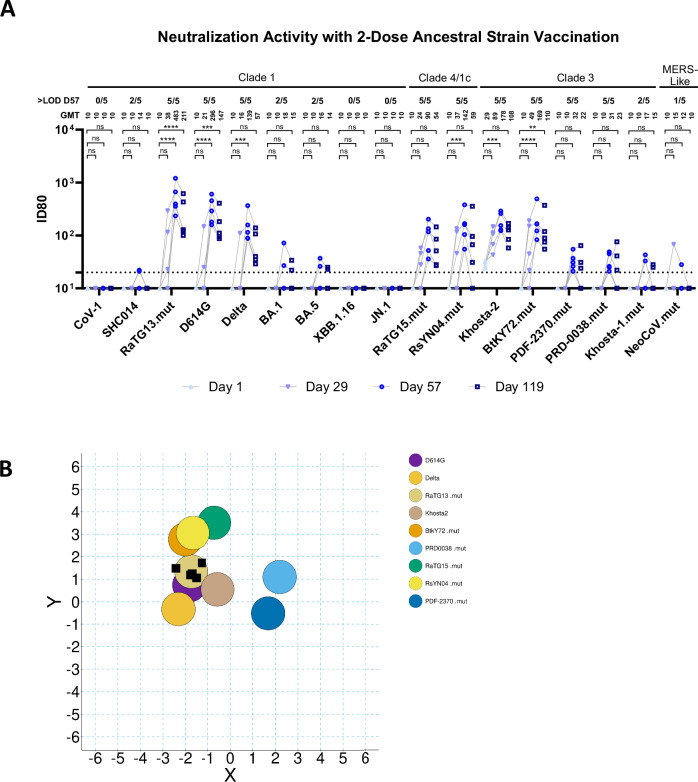
Neutralization Activity with 2-Dose Ancestral Strain Vaccination. **A** Serum neutralizing titers (ID80) of the DMID 20-0003 samples (*N* = 5) at days 1, 29 (4 weeks post-vaccination), 57 (4 weeks post-second vaccination), and 119 against the indicated pseudoviruses. Geometric mean ID80 titers (GMT) are shown along with the number of samples with detectable titers at day 57. The dotted line represents the assay limit of detection (LOD) of 20. Data below the LOD were assigned a value of 10. Two-way ANOVA was performed with Tukey’s multiple comparison test for statistical assessment; only comparisons to day 1 are indicated: * *p* < 0.05, ** *p* < 0.01, *** *p* < 0.001, **** *p* < 0.0001, ns is non-significant. Pseudotyped viruses with a ‘.mut’ suffix were made with spike protein sequences containing the mutations listed in Supplementary Table 1.** B** Antigenic map from individuals in DMID 20-0003 with two doses of prior ancestral vaccination at Day 57. Antigens are denoted by circles, sera by black squares. Only antigens against which at least 3 individuals had detectable titers were included in this map. Each unit of antigenic distance between serum-antigen pairs on the map corresponds to a fold-drop in neutralization titer for that serum against the antigen in question relative to the antigen against which the serum has the highest neutralization titer.

To assess if the original vaccination schedule against the Wuhan (i.e., ancestral) strain of SARS-CoV-2 elicited broad responses against clade 3 and 4 sarbecoviruses, we tested neutralization using our pseudovirus panel against sera collected in 2020 from five volunteers in the DMID 20-0003 COVID-19 vaccine clinical trial. None of the individuals had been infected with SARS-CoV-2 before the trial or during the time the sera were collected. Moderna’s mRNA-1273 vaccine was administered on study days 1 and 29 (50 µg doses), and we assessed sera collected on the days of administration as well as on days 57 and 119 of the study. Consistent with previous results^12^, we found there was no neutralizing activity in the day 1 sera, and a weak titer was measurable by day 29 after initial vaccination in three of the five individuals against SARS-CoV-2 D614G (Fig. 1A). By day 57, four weeks after the second vaccination, all five volunteers exhibited robust sera neutralizing activity against the D614G pseudovirus. Geometric mean ID80 titers (GMT) moderately decreased from day 57 (296) to day 119 (147), consistent with previous findings^12^. Within clade 1b, RaTG13 mutant and the Delta variant titers were within 3-fold of D614G at day 57 (GMT of 483 and 139 vs 296, respectively). Little to no neutralization was observed against the Omicron variants tested, as well as against clade 1a SARS-CoV-1 and SHC014. Notably, we observed moderate neutralization at day 57 in all 5 individuals against clade 4 pseudovirus mutants of RaTG15 and RsYN04 at day 57 (GMT of 90 and 142, respectively). While titers against the clade 3 sarbecoviruses were lower than for SARS-CoV-2 D614G, all 5 individuals had detectable responses above baseline at day 57 against Khosta-2 as well as BtKY72, PDF-2370, and PRD-0038 mutant pseudoviruses (GMT of 178, 169, 32, and 31, respectively). Only low titers were observed in 2 individuals against the Khosta-1 mutant, and only 1 individual had a detectable titer against the NeoCoV mutant at day 57 (Fig. 1A). Antigenic maps constructed from day 57 titers indicated that the Delta variant was at least twice as close to D614G as several clade 3 and 4 viruses, with a 2- to 5-fold difference in neutralization titer between D614G and Delta, compared to a 9- to 24-fold difference between D614G and PRD-0038, and a 10- to 34-fold difference between D614G and PDF-2370 (Fig. 1B, Supplementary Fig. 1).

### Neutralization activity pre- and post-omicron infection

**Fig. 2:**
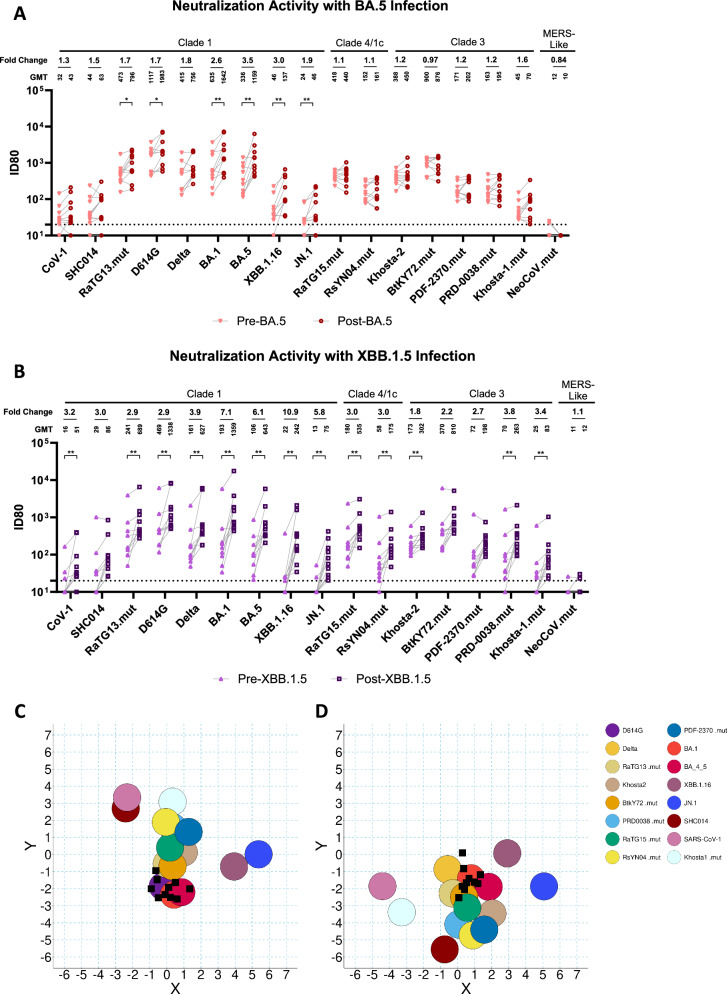
Neutralization Activity with Omicron Infection. Serum neutralizing titers (ID80) of the DMID 22-0004 samples pre- and post-infection with **A** BA.5 (*N* = 10) or **B** XBB.1.5 (*N* = 10) against the indicated pseudoviruses. Geometric mean ID80 titers (GMT) are shown along with fold change values. The dotted line represents the assay limit of detection (LOD) of 20. Data below the LOD were assigned a value of 10. Two-tailed Wilcoxon matched pairs signed rank test was used for statistical assessment; only comparisons to pre-infection are indicated: * *p* < 0.05, ** *p* < 0.01, *** *p* < 0.001, **** *p* < 0.0001. Pseudotyped viruses with a ‘.mut’ suffix were made with spike protein sequences containing the mutations listed in Supplementary Table 1. Antigenic pseudo-map from individuals in DMID 22-0004 after infection with (**C**) BA.5 or (**D**) XBB.1.5 antigens are denoted by circles, sera by squares. Only antigens against which at least 3 individuals had detectable titers were used to construct this map. Triangulation of D614G with respect to the other antigens is shown in Supplementary Fig. 1. Each unit of antigenic distance between serum-antigen pairs on the map corresponds to a fold-drop in neutralization titer for that serum against the antigen in question relative to the antigen against which the serum has the highest neutralization titer.

Given the rapid evolution of new SARS-CoV-2 variants and changes to the vaccination schedule, we next sought to determine if a more current immunological landscape still conferred neutralization against clade 3 and 4 sarbecoviruses, as well as if infection with recent Omicron variants would further enhance neutralization potency. We evaluated sera from a subset of 20 individuals enrolled in the DMID 22-0004 trial that had documented infection in 2022 (annotated as BA.5) or in 2023 (annotated as XBB.1.5), irrespective of collection timepoint or trial enrollment arm (Supplementary Data 1). The scheduled collections immediately preceding and proceeding documented infection were evaluated against our pseudovirus panel. Serum collection dates ranged from 54 to 32 days for BA.5 pre-infection and 61 to 17 days for XBB.1.5 pre-infection. Dates ranged from 8 to 51 days for BA.5 post-infection and 15 to 59 days for XBB.1.5 post-infection (Supplementary Data 1). Although consisting of different timepoints and study arms, baseline GMTs against most clade 1, 3, and 4 pseudoviruses were comparable at pre-infection in the DMID 22-0004 samples as compared to peak DMID 20-0003 titers (Supplementary Fig. 2). Infection with BA.5 (Fig. 2A) or XBB.1.5 (Fig. 2B) caused an increase in GMT by 3.5- and 10.9-fold against BA.5 and XBB.1.16, respectively. While infection with BA.5 significantly increased titers against only the RaTG13 mutant, D614G, and the Omicron variants, XBB.1.5 infection significantly increased titers for SARS-CoV-1 and across all clade 1b and 4 pseudoviruses. Although the GMT for clade 3 pseudoviruses increased after XBB.1.5 infection, only the increases in Khosta-2, PRD-0038.mut and Khosta-1.mut were statistically significant (Fig. 2B). Neither post-infection events resulted in a change in titers against the NeoCoV mutant pseudovirus. We generated a set of antigenic pseudo-maps from post-infection titers, irrespective of prior exposure histories. Following BA.5 infection (Fig. 2C and Supplementary Fig. 1C and D), BtKY72 has a 2- to 3-fold difference from D614G, while RaTG15 and Khosta-2 have a 4- to 6-fold difference from D614G. PRD-0038, RsYN04, and PDF-2370 have a 10- to 17-fold difference from D614G but are closer to D614G than XBB.1.16 is. Khosta-1 has a 26- to 41-fold difference from D614G so is slightly closer to D614G than SARS-CoV-1 is and closer than JN.1 is (Supplementary Fig. 1). In the post-XBB.1.5 infection antigenic pseudo-map (Fig. 2D and Supplementary Fig. 1E and F), BA.1 is the closest antigen with a 1- to 2-fold difference from D614G. BtKY72 and RaTG15 have a 2- to 3-fold difference from D614G, similar in distance to Delta and BA.5. Khosta-2, PRD-0038, RsYN04, and PDF-2370 are within a 5- to 13-fold difference from D614G, in a similar range as XBB.1.16. Khosta-1 and SHC014 were both within a 15- to 24-fold difference from D614G, slightly closer than SARS-CoV-1, which was within a 25- to 36-fold difference from D614G as well as JN.1 which ranged from a 22- to 35-fold difference from D614G. In general, the antigenic pseudo-map generated from post XBB.1.5 infections shows that more clade 3 and 4 viruses clustered closer to D614G than those generated from post BA.5 infection sera, and some clade 3 and 4 viruses clustered closer to D614G than others.

### Neutralization activity against wild type clade 3 and 4 pseudoviruses

**Fig. 3:**
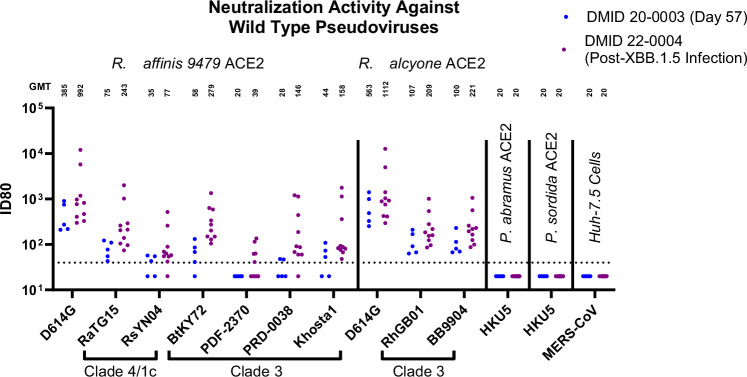
Neutralization Activity Against Wild Type Clade 3 and 4 Pseudoviruses. Serum neutralizing titers (ID80) of the DMID 20-0003 samples at Day 57 (*N* = 5) and DMID 22-0004 samples post-infection with XBB.1.5 (*N* = 10) against the indicated pseudoviruses. Assays were performed in 293T cells stably expressing *R. affinis 9479* ACE2 (Accession: QMQ39227.1), *R. alcyone* ACE2 (Accession: ALJ94035.1), *P. abramus* ACE2 (Accession: ACT66266.1), or *P. sordida* ACE2 (Accession: NWI93316.1). The MERS-CoV pseudovirus assay was performed using Huh-7.5 cells that contain endogenous DPP4. Geometric mean ID80 titers (GMT) are shown. The dotted line represents the assay limit of detection (LOD) of 40. Data below the LOD were assigned a value of 20.

As the RBD mutations introduced into the pseudoviruses were selected to facilitate higher entry via human ACE2 and may not necessarily be reflective of mutations that would occur in the event of zoonotic spillover, we evaluated if neutralization would be observed using wild type clade 3 and 4 spikes in 293 T cells stably expressing ACE2 orthologs from bat species *Rhinolophus affinis* or *Rhinolophus alcyone* (Supplementary Table 1). This also allowed us to test two additional clade 3 pseudoviruses, RhGB01 and BB9904, for which we did not identify mutations that facilitated the usage of human ACE2 in our lentivirus system. We also evaluated an HKU5 pseudovirus in 293 T/17 cells transiently expressing ACE2 from *Pipistrellus abramus* or *Pitta sordida*, as HKU5 is a merbecovirus that has recently been demonstrated to use ACE2 for entry^13–15^. As shown with human ACE2 (Fig. 1A), vaccination with only 2 doses of mRNA-1273 was sufficient to measure ID80 neutralization against clade 3 and 4 sarbecoviruses (Fig. 3), including against RhGB01 and BB9904, albeit not PDF-2370. As shown with the mutants (Fig. 2B), samples collected post-XBB.1.5 infection showed robust GMT against all wild-type clade 3 and 4 pseudoviruses tested (Fig. 3). No samples evaluated had neutralization against HKU5 or MERS pseudoviruses.

## Discussion

Our data suggest that the SARS-CoV-2 vaccination strategy implemented at the start of the COVID-19 pandemic elicited antibodies that cross-reacted with clade 3 and 4 sarbecoviruses. The exposure to new strains through vaccination and infection, as the pandemic evolved, matured responses that continue to be both cross-reactive and potent. While genetic distance of SARS-CoV-2 variants from the ancestral strain has been correlated with worsened vaccine efficacy and antibody titers^16–18^, our study suggests that genetic distance may not be predictive across sarbecovirus clades. Across the cohorts we interrogated, JN.1, SARS-CoV-1, and Khosta-1 sarbecoviruses had among the furthest antigenic map distances from SARS-CoV-2 D614G. However, they vary from ancestral SARS-CoV-2 by 9%, 26%, and 30% in RBD amino acid homology, respectively, whereas BtKY72 which had neutralization titers within 2- to 3-fold from D614G across cohorts diverges by 27% in RBD amino acid homology compared to ancestral SARS-CoV-2 (Supplementary Fig. 3). The RBD homology from SARS-CoV-1 to JN.1, Khosta-1, and BtKY72 differ by 30%, 26%, and 26%, respectively. Therefore, clade 1a and clade 3 viruses have similar RBD homology to each other as they do to clade 1b, highlighting that conservation of specific neutralizing epitopes is not necessarily reflected in cross-clade phylogeny comparisons. Therefore, our study reinforces the need for active antigenic mapping and model building with new variants and emerging species of sarbecoviruses, especially where different evolutionary pressures exist to extend tropism versus immune escape^19,20^.

Our results align well with previous studies that have identified neutralization or binding responses against at least one clade 3 or 4 sarbecovirus in vaccinated mice^6,21^ as well as isolation of monoclonal antibodies that are able to broadly neutralize recent SARS-CoV-2 variants and clade 3 and 4 sarbecoviruses^22–25^. Similarly, previous studies have indicated that vaccination and infection with SARS-CoV-2 can elicit a response against SARS-CoV-1 and other clade 1a sarbecoviruses^25–28^. Our findings profile the breadth of these responses and suggest that these functional cross-clade responses have been boosted in the current landscape of repeated vaccinations and Omicron lineage infections. While we did not note statistically significant increases in GMT against clade 3 and 4 sarbecoviruses after BA.5 infection, we did observe notable changes after XBB.1.5 infection. Given potential differences in immunogenicity between BA.5 and XBB.1.5 infections, differences in prior vaccination histories between these cohorts, and lower pre-infection titers observed in the XBB.1.5 cohort relative to the BA.5 cohort, we cannot determine why XBB.1.5 infection led to a more robust increase in neutralization for certain pseudoviruses. Future work to isolate and profile cross-clade-reactive B cells across these cohorts may yield such insights.

Our study cannot determine whether observed titers are protective or necessarily indicative of risk. In addition, because we measured only pseudovirus entry inhibition and not binding levels, Fc-mediated functions, or T-cell activity, our conclusions are limited to neutralizing antibody responses. However, given neutralization titer is a well-established correlate of protection for SARS-CoV-2^7–10^, the titers identified here are likely indicative of some level of protective immunity. Furthermore, the fact that the antigenic distances between the ancestral variant and several clade 3 and 4 viruses on the post-Omicron infection antigenic pseudo-map are comparable to the distances between the ancestral variant and other SARS-CoV-2 strains indicates that immunity against SARS-CoV-2 strains may also extend to those sarbecoviruses. By extension, zoonotic spillover of clade 3 and 4 sarbecoviruses may require mutations not only to adopt use of the human ACE2 receptor but also to evade existing immunity developed through repeated SARS-CoV-2 exposures. Therefore, clade 3 and 4 strains may have a lower barrier for using human ACE2 for infection but have a higher barrier to overcome existing immunity. Conversely, coronaviruses such as HKU5 may have a higher barrier to adopt efficient use of human ACE2, but population level immunity may not need to be overcome. Thus, population level decisions concerning pandemic preparedness against sarbecoviruses should consider both their immunological escape potential as well as current levels of immunity.

## Methods

### Clinical cohorts

DMID 20-0003 (NCT04283461) was a first-in-human phase 1 clinical trial in healthy adults to evaluate the safety and immunogenicity of mRNA-1273 with enrollment between March and June 2020. To evaluate immunity after vaccination with the primary vaccine schedule, collections from five individuals were randomly selected from Cohort 10 of DMID 20-0003 (15 adults aged 18-55 that received two 50 mcg doses of mRNA-1273), and serum from days 1, 29, 57, and 119 were evaluated. Details regarding the trial protocol, conduct, and outcomes have been previously published^12,29^.

DMID 22-0004 (COVAIL Study, NCT05289037) was an adaptive phase 2 open-label clinical trial in healthy adults to compare boosting with ancestral and variant SARS-CoV-2 spike protein(s) (Beta, Delta and Omicron BA.1), alone or in combination, using both mRNA vaccines (Moderna and Pfizer BioNTech mRNA), and recombinant protein vaccine (Sanofi AS03-adjuvanted) on the breadth, magnitude, and durability of immunogenicity. The trial consisted of multiple independently designed stages, each of which enrolled sequentially based on vaccine selection in prior stages and the availability of new variant vaccine products. The trial was reviewed and approved by the Advarra institutional review board and monitored by an independent data and safety monitoring board. Details regarding the trial protocol, conduct, and outcomes have been previously published^30^.

Collections from 10 individuals with documented BA.5 and 10 individuals with documented XBB.1.5 infections during the course of the study were selected, and serum from the nearest collection before and after infections was evaluated. Infection with SARS-CoV-2 was either self-reported and confirmed by detection of nucleoprotein antibodies and/or PCR or by only the detection of nucleoprotein antibodies and/or PCR in samples collected as a normal study timepoint. The infecting variant was assigned based on the time of infection and the most common circulating variant. To ensure our analysis was sufficiently powered to assess neutralization activity pre- and post-infection, collections were randomly selected irrespective of enrollment arm. Data regarding the infection and collection dates, as well as enrollment groups shared by the study sponsor, are listed in Supplementary Data 1.

### Pseudovirus Creation and Neutralization Assays

Spike-containing lentiviral pseudovirions were produced by co-transfection of packaging plasmid pCMVΔR8.2, firefly luciferase-encoding plasmid pHR’ CMV-Luc, and Spike plasmids from sarbecoviruses into 293T cells (ATCC #CRL-11268) using Lipofectamine 3000 transfection reagent (ThermoFisher) as previously described^31^. Plasmid synthesis was performed by GenScript (Piscataway, NJ). Sarbecovirus spike accession IDs and mutants used are listed in Supplementary Table 1. Stable cell lines were created by transfecting 293T cells (ATCC #CRL-11268) using Lipofectamine 3000 transfection reagent and plasmids containing the indicated ACE2 under a CMV promoter and with a C-terminal flag-tag. After transfection, cells were cultured in puromycin-containing media. All cell lines are regularly tested for mycoplasma and have consistently tested negative.

A pre-titrated dose of pseudovirus was incubated with heat-inactivated (56 °C for 60 minutes) serum samples in duplicate at eight serial dilutions in 96-well flat-bottom tissue culture plates (ThermoFisher, #137103) for 1 h at 37 °C prior to adding 293T-ACE2 cells (cells provided by Dr. Michael Farzan). One set of wells received cells and virus (virus control), and another set of wells received cells only (background control). Luminescence was measured after 66-72 hours of incubation using Britelite Plus luciferase reagent (PerkinElmer, #6066769). The luminescence signal (relative light unit (RLU)) was measured using a Molecular Devices Paradigm or i3x multimode reader. Neutralization titers are the inhibitory dilution of serum samples at which RLUs were reduced by 80% (ID80) compared to virus control wells after subtraction of background RLUs.

### Phylogeny and antigenic cartography

Phylogeny trees were created using NCBI tree viewer with accession numbers listed in Supplementary Table 1. Sequences were mapped using a fast minimum evolution tree method with Grishin general distance relative to ancestral SARS-CoV-2 spike residues 331-531. Alignments were performed using the NCBI multiple sequence alignment viewer version 1.26.0. The RBD homology percentage heatmap was generated in Geneious Prime v2025.2.1 using the MUSCLE multiple sequence alignment algorithm with default parameters. Pairwise percent identity values were calculated and visualized as a heatmap.

Publicly available antigenic map and plotting code from Wang et al.^32^ was used to construct antigenic maps. All antigenic cartography analyses were performed in R (version 4.3.0) and analyzed with the R package racmacs (version 1.2.9, compiled with gcc 11.3.0). Triangulation and noisy bootstrap maps are shown in Supplementary Fig. 1. For the noisy bootstrap maps, random noise with a standard deviation of 0.2878 on a log2 scale was used following the approach of Wang et al.^32^. Confidence intervals for fold differences were obtained from 95 percent quantiles from the noisy bootstrapping. Maps were plotted using the R package ggplot2 (version 3.5.0). Antigenic pseudo-maps in Fig. 2 were constructed and analyzed using the identical procedure as traditional antigenic maps, such as those in Fig. 1, except that we do not interpret these maps as traditional maps since the prior exposure histories of the sera before infection are unknown.

### Bioethics statement

We characterized the cell entry of pseudotyped sarbecovirus spikes and spike mutants with point mutations that increase cell entry via human ACE2 using non-replicative lentiviral particles. All work was performed with prior approvals in a BSL-2 laboratory, as our experiments involve only the spike proteins of the listed sarbecoviruses, which cannot replicate. Neither live virus nor a replicative viral system was used during the course of this study or is planned for future use. Furthermore, the mutations or mutational strategy utilized here has been previously described^3,11^.

### Statistical analysis

The neutralization curve fit was generated on a NAB analysis module on the Labkey web-based server to calculate ID80 values. Undetectable titers were set to half the limit of detection for graphing and analysis (LOD/2). Statistical significance was evaluated using a two-way ANOVA with Tukey’s multiple comparison test across vaccination timepoints, and the Wilcoxon matched-pairs signed rank test was used to compare pre- and post-infection in GraphPad Prism v.10 software. Values used to make all figures in this manuscript are included in the source data file.

### Ethics statement

All participants provided written informed consent before enrollment into the DMID 20-0003 (NCT04283461) and DMID 22-0004 (COVAIL Study, NCT05289037) trials. Trial protocols were reviewed and approved by the Advarra institutional review board. Research performed in this study was conducted with prior approval by the trial sponsors and without access to identifiable private information.

### Reporting summary

Further information on research design is available in the Nature Portfolio Reporting Summary linked to this article.

## Supplementary information

Supplementary Information

Description of Additional Supplementary File

Supplementary Data 1

Reporting summary

Transparent Peer Review file

## Source data

source data

## Data Availability

Source data are provided with this paper in the source data file. Any additional information required to reanalyze the data reported in this paper is available from the lead contacts upon request. Source data are provided with this paper.

## Code availability

This paper does not report original code.

## Materials availability

All reagents generated in this study are available from the lead contacts upon completion of a materials transfer agreement.
