# Supplementary material for: SARS-CoV-2 vaccination and infection elicit cross-neutralizing responses against clade 3 and 4 sarbecoviruses
Source: Nat Commun. 2026 Apr 16;17:5245. doi: 10.1038/s41467-026-71662-y (PMC13260318; doi:10.1038/s41467-026-71662-y)
Supplement: Supplementary file 1 — Supplementary Information [file 41467_2026_71662_MOESM1_ESM.pdf]

Supplementary Information

SARS-CoV-2 Vaccination and Infection Elicit Cross-Neutralizing Responses against Clade 3 and 4 Sarbecoviruses

Supplementary Figures 1-3

Supplementary Table 1

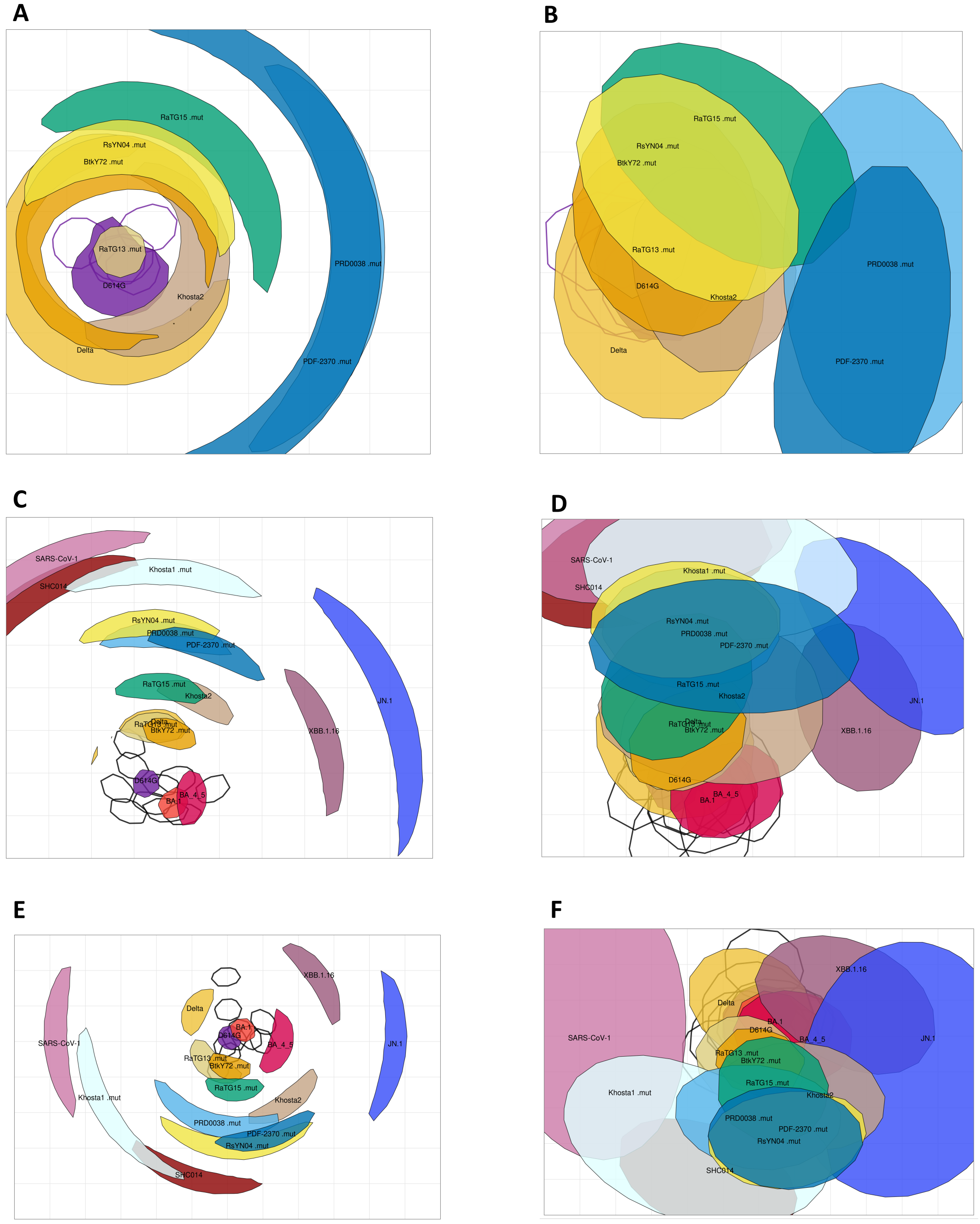

Supplementary Figure 1: Antigenic landscape triangulation and bootstrap maps
Triangulation/coordination confidence intervals for DMID 20-0003 vaccinee neutralization titers (A), DMID 22-0004 BA.5 post-infection titers (C), and DMID 22-0004 XBB.1.5 post-infection titers (E). Each shape denotes the region of the map a point can occupy without the total map stress increasing by more than 1 unit of antigenic distance.

Noisy bootstrap maps for DMID 20-0003 vaccinee neutralization titers (B), DMID 22-0004 BA.5 post-infection titers (D), and DMID 22-0004 XBB.1.5 post-infection titers (F) that incorporates measurement error in titer measurements for the neutralization assay. For each of 100 bootstrap replicates, random noise is added to the titer data to simulate variation due to measurement error, and a replicate antigenic map is reconstructed. Antigen shapes represent possible positions across all replicate maps.

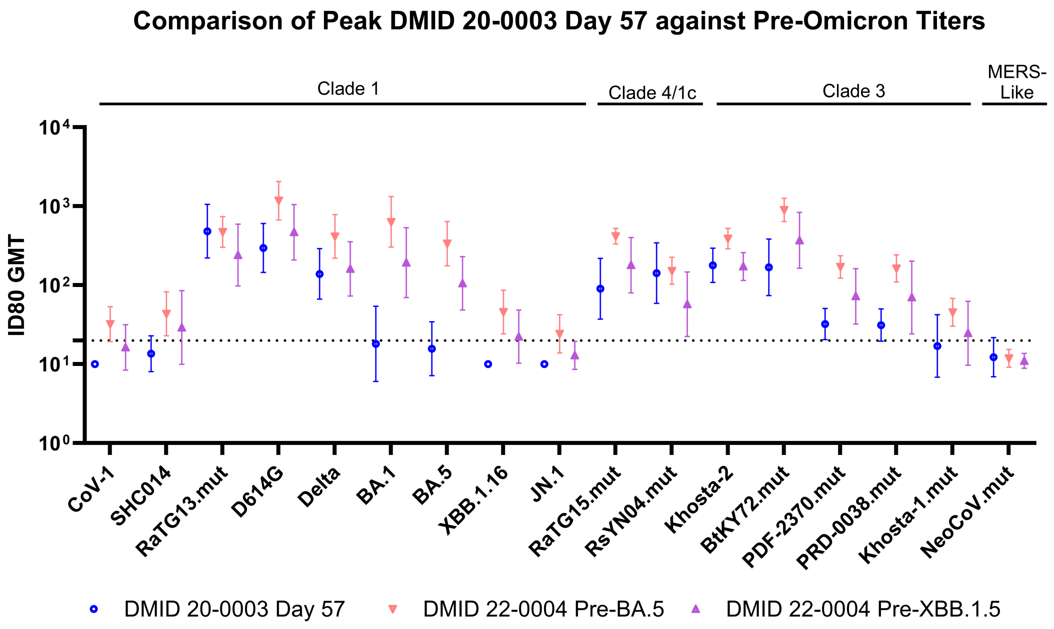

Supplementary Figure 2: Comparison of DMID 20-0003 Day 57 against pre-Omicron titers
Serum neutralizing titers (ID80) of the DMID 20-0003 samples at Day 57 (N=5) and DMID 22-0004 samples pre-infection with BA.5 (N=10) or XBB.1.5 (N=10) against the indicated pseudoviruses. Geometric mean ID80 titers (GMT) are shown with 95% confidence intervals. No statistically significant differences were noted between groups for each pseudovirus by 2-way ANOVA. The dotted line represents the assay limit of detection (LOD) of 20. Data below the LOD were assigned a value of 10.

A

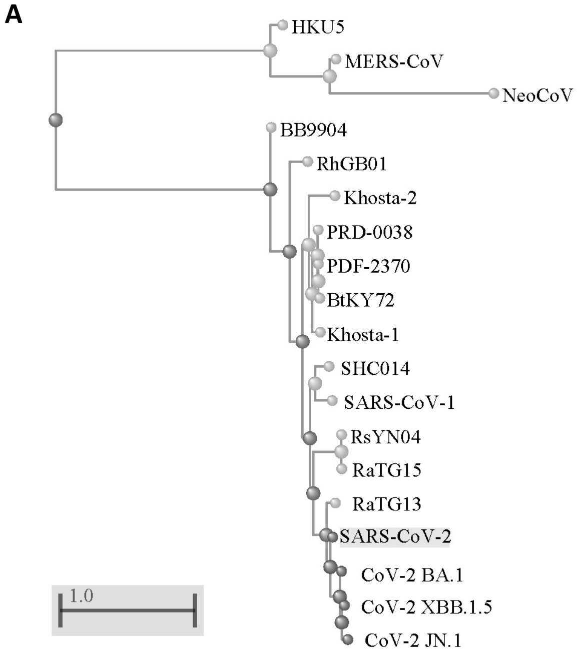

B

	Clade 1b					Clade 4/1c		Clade 1a		Clade 3							MERS-Like		
	SARS-CoV-2	BA.5	XBB.1.5	JN.1	RaTG13	RaTG15	RsYN04	SARS-CoV-1	SHC014	Khosta-1	BtKY72	PDF-2370	PRD-0038	Khosta-2	RhGB01	BB9904	NeoCoV	MERS-CoV	HKU5
**SARS-CoV-2**		91.5	89.1	86.6	89.6	71.6	71.6	74.1	77.1	70.3	73.3	73.8	73.8	67.8	67.5	65.8	18.8	25.3	16.6
**BA.5**			95.5	93.0	85.6	68.2	68.2	70.6	73.6	66.3	69.3	69.8	69.8	65.3	63.5	63.4	20.3	25.3	15.1
**XBB.1.5**				93.5	85.1	67.7	67.7	70.6	75.6	66.8	68.8	69.3	69.3	63.9	63.1	62.9	19.8	24.2	15.1
**JN.1**					82.1	67.2	67.2	70.5	74.5	66.3	68.3	68.8	68.8	63.9	62.6	63.4	20.4	25.3	15.2
**RaTG13**						71.1	71.1	76.1	77.1	71.8	72.3	72.8	72.8	66.8	66.0	64.9	18.3	25.3	15.6
**RaTG15**							99.5	69.2	70.1	71.2	70.5	71.4	71.0	65.2	64.0	68.7	15.3	28.3	14.9
**RsYN04**								69.2	70.1	71.2	70.5	71.4	71.0	65.7	64.0	68.7	15.3	28.3	14.9
**SARS-CoV-1**									81.5	74.3	74.3	74.3	74.8	70.3	68.0	68.3	17.9	24.2	16.2
**SHC014**										73.8	75.2	75.7	75.7	68.8	67.0	69.8	18.9	25.3	15.7
**Khosta-1**											87.5	88.9	89.0	79.3	73.0	78.8	16.3	26.0	14.9
**BtKY72**												98.5	98.0	78.5	74.1	80.5	15.8	27.0	15.7
**PDF-2370**													98.5	78.9	74.5	80.4	16.3	27.0	15.8
**PRD-0038**														78.5	74.1	79.5	16.3	27.0	15.7
**Khosta-2**															71.0	73.2	14.8	27.0	15.8
**RhGB01**																71.5	14.8	28.0	16.3
**BB9904**																	17.2	30.0	16.9
**NeoCoV**																		49.0	34.5
**MERS-CoV**																			67.3
**HKU5**																			

C

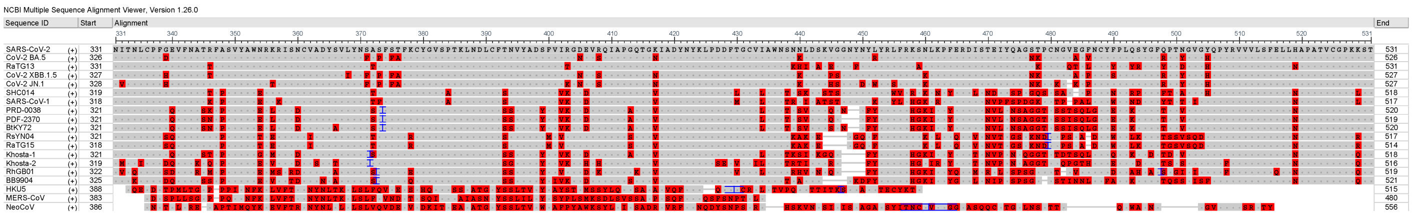

Supplementary Figure 3: Phylogenetic comparison of RBD sequences
(A) Phylogeny tree of RBDs using the accession numbers listed in Supplementary Table 1.
(B) Pairwise percent identity values of the RBD regions calculated and visualized as a heatmap.
(C) RBD alignments using the accession numbers listed in Supplementary Table 1.

Supplementary Table 1: Pseudovirus accession IDs, mutations, and entry levels

Sabrcoviruses Name	GenBank Accession Number	Presumed Region	RBD Clade Designation	Pseudovirus Concentrated?	Max Signal (RLU) in Human ACE2 Cells at Neat Dilution	Mutation(s) Added	Max Signal (RLU) in Human ACE2 Cells at Neat Dilution (After Spike Mutations Introduced)	ACE2 Orotholog(s) Used	Pseudovirus Concentrated?	Max Signal (RLU) in ACE2 Ortholog Over-Expressing Cells at Neat Dilution
SARS-CoV-2 D614G	QHR63290.2	N/A	1b	No	1,272,375	D614G	N/A	*R. affinis* 9479; *R. alcyone*	No	27,269; 12,188
SARS-CoV-2 Delta	N/A	N/A	1b	No	1,068,780	N/A	N/A	N/A	N/A	N/A
SARS-CoV-2 BA.1	N/A	N/A	1b	No	104,741	N/A	N/A	N/A	N/A	N/A
SARS-CoV-2 BA.5	N/A	N/A	1b	No	1,663,899	N/A	N/A	N/A	N/A	N/A
SARS-CoV-2 XBB.1.16	N/A	N/A	1b	No	1,279,775	N/A	N/A	N/A	N/A	N/A
SARS-CoV-2 JN.1	N/A	N/A	1b	No	1,824,268	N/A	N/A	N/A	N/A	N/A
RaTG13	QHR63300.2	China	1b	No	2,705	D501V	29,139	N/A	N/A	N/A
SARS-CoV-1 (Urbani)	AAP13441.1	China/Vietnam	1a	No	4,766,148	N/A	N/A	N/A	N/A	N/A
SHC014	QJE50589.1	China	1a	No	34,674	N/A	N/A	N/A	N/A	N/A
RaTG15	UFP05053.1	China	4 (aka 1c)	No	< 1,000	L476Y,T481W ,S485G,Q486V,D487G	98,681	*R. affinis* 9479	No	7,833
RsYN04	QWN56242.1	China	4 (aka 1c)	No	< 1,000	L479Y,T484W,S488G,Q489V,D490G	21,754	*R. affinis* 9479	10X	5,116
Khosta-2	QVN46569.1	Russia	3	10X	30,020	N/A	N/A	N/A	N/A	N/A
Khosta-1	QVN46559.1	Russia	3	No	< 1,000	K480Y,D483G,T485W	21,609	*R. affinis* 9479	5X	11,888
BtKY72	APO40579.1	Kenya	3	No	< 1,000	K482Y,T487W	36,120	*R. affinis* 9479	No	35,794
PRD-0038	QTJ30153.1	Rwanda	3	No	< 1,000	K482Y,T487W	24,181	*R. affinis* 9479	No	19,148
PDF-2370	QTJ30144.1	Uganda	3	No	< 1,000	K481Y,T486W	266,308	*R. affinis* 9479	No	34,842
RhGB01	QYC92806.1	United Kingdom	3	No	< 1,000	N/A	N/A	*R. alcyone*	5X	26,805
BB9904	ALJ94036.1	Bulgaria	3	No	< 1,000	N/A	N/A	*R. alcyone*	No	352,953
HKU5	YP_001039962.1	Hong Kong	N/A	No	< 1,000	N/A	N/A	*P. abramus; P. sordida*	No	25,059; 10,662
NeoCoV	AGY29650.2	sub-Saharan Africa	N/A	No	< 1,000	T510F	117,616	N/A	N/A	N/A
MERS-CoV	QGV13484.1	Saudi Arabia	N/A	No	N/A	N/A	N/A	N/A (used Huh-7.5 cells)	10X	6,044

RBD, receptor binding domain; RLU, relative light units; ACE2, angiotensin converting enzyme 2; N/A, not applicable; aka, also known as
Strain names, where applicable, are listed parenthetically.
