# Supplementary material for: SARS-CoV-2 vaccination and infection elicit cross-neutralizing responses against clade 3 and 4 sarbecoviruses
Source: Nat Commun. 2026 Apr 16;17:5245. doi: 10.1038/s41467-026-71662-y (PMC13260318; doi:10.1038/s41467-026-71662-y)
Supplement: Supplementary file 2 — Description of Additional Supplementary File [file 41467_2026_71662_MOESM2_ESM.pdf]

Description of Additional Supplementary Files

Supplementary Data 1:
Sample study arm and infection details
