# Supplementary material for: SARS-CoV-2 vaccination and infection elicit cross-neutralizing responses against clade 3 and 4 sarbecoviruses
Source: Nat Commun. 2026 Apr 16;17:5245. doi: 10.1038/s41467-026-71662-y (PMC13260318; doi:10.1038/s41467-026-71662-y)
Supplement: Supplementary file 4 — Reporting summary [file 41467_2026_71662_MOESM4_ESM.pdf]

nature portfolio

Corresponding author(s): L. Serebryannyy and D. Douek

Last updated by author(s): Mar 4, 2026

Reporting Summary

Nature Portfolio wishes to improve the reproducibility of the work that we publish. This form provides structure for consistency and transparency in reporting. For further information on Nature Portfolio policies, see our Editorial Policies and the Editorial Policy Checklist.

Statistics

For all statistical analyses, confirm that the following items are present in the figure legend, table legend, main text, or Methods section.

n/a	Confirmed	
☐	☒	The exact sample size (*n*) for each experimental group/condition, given as a discrete number and unit of measurement
☐	☒	A statement on whether measurements were taken from distinct samples or whether the same sample was measured repeatedly
☐	☒	The statistical test(s) used AND whether they are one- or two-sided<br>*Only common tests should be described solely by name; describe more complex techniques in the Methods section.*
☐	☒	A description of all covariates tested
☐	☒	A description of any assumptions or corrections, such as tests of normality and adjustment for multiple comparisons
☐	☒	A full description of the statistical parameters including central tendency (e.g. means) or other basic estimates (e.g. regression coefficient) AND variation (e.g. standard deviation) or associated estimates of uncertainty (e.g. confidence intervals)
☐	☒	For null hypothesis testing, the test statistic (e.g. *F*, *t*, *r*) with confidence intervals, effect sizes, degrees of freedom and *P* value noted<br>*Give P values as exact values whenever suitable.*
☒	☐	For Bayesian analysis, information on the choice of priors and Markov chain Monte Carlo settings
☒	☐	For hierarchical and complex designs, identification of the appropriate level for tests and full reporting of outcomes
☒	☐	Estimates of effect sizes (e.g. Cohen's *d*, Pearson's *r*), indicating how they were calculated

Our web collection on statistics for biologists contains articles on many of the points above.

Software and code

Policy information about availability of computer code

Data collection	Luminescence, measured in relative light units, was measured using a Molecular Devices luminometer.
Data analysis	The neutralization curve fit was generated on a NAB analysis module on the Labkey web-based server with five-parameter nonlinear regression. Neutralizing antibody titers are expressed as the reciprocal of the serum dilution required to reduce RLU by 80% and are reported as inhibition dosage.<br>Publicly available antigenic map and plotting code from Wang et al.Cell Host Microbe. 2022 was used to construct antigenic maps. All antigenic cartography analyses were performed using R (version 4.3.0) and analyzed using the R package racmacs (version 1.2.9 with gcc compiler 11.3.0).

For manuscripts utilizing custom algorithms or software that are central to the research but not yet described in published literature, software must be made available to editors and reviewers. We strongly encourage code deposition in a community repository (e.g. GitHub). See the Nature Portfolio guidelines for submitting code & software for further information.

Data

Policy information about availability of data

All manuscripts must include a data availability statement. This statement should provide the following information, where applicable:

- Accession codes, unique identifiers, or web links for publicly available datasets
- A description of any restrictions on data availability
- For clinical datasets or third party data, please ensure that the statement adheres to our policy

The trial dataset will be available to appropriate academic parties on request from the corresponding author, in accordance with the data sharing policies of trial Sponsors with input from the investigator group subject to submission of a suitable study protocol and analysis plan. Source data are provided with this paper.

Research involving human participants, their data, or biological material

Policy information about studies with human participants or human data. See also policy information about sex, gender (identity/presentation), and sexual orientation and race, ethnicity and racism.

Reporting on sex and gender	Information on sex and gender were collected by self-reporting during the course of the clinical trials. This information was not used in the context of the exploratory serological analyses in this report.
Reporting on race, ethnicity, or other socially relevant groupings	Information on race/ethnicity were collected by self-reporting during the course of the clinical trials. The information was not used in the context of the exploratory serological analysis in this report.
Population characteristics	Population characteristics for DMID 20-0003 (NCT04283461) were described in Jackson et al NEJM 2020.<br>Population characteristics for DMID 22-0004 (COVAIL Study, NCT05289037) were described in Branche et al. Nat Med 2023.
Recruitment	Recruitment for DMID 20-0003 (NCT04283461) was described in Jackson et al NEJM 2020.<br>Recruitment for DMID 22-0004 (COVAIL Study, NCT05289037) was described in Branche et al. Nat Med 2023.
Ethics oversight	NCT04283461 and NCT05289037 were reviewed and approved by the Advarra institutional review board, which functioned as a single board and was overseen by an independent safety monitoring committee. All participants provided written informed consent before enrollment. Research performed in this report was conducted with prior approval by the trial sponsors and without access to identifiable private information.

Note that full information on the approval of the study protocol must also be provided in the manuscript.

Field-specific reporting

Please select the one below that is the best fit for your research. If you are not sure, read the appropriate sections before making your selection.

☒ Life sciences ☐ Behavioural & social sciences ☐ Ecological, evolutionary & environmental sciences

For a reference copy of the document with all sections, see nature.com/documents/nr-reporting-summary-flat.pdf

Life sciences study design

All studies must disclose on these points even when the disclosure is negative.

Sample size	For DMID 20-0003, a single arm best representing the initial prescribed COVID-19 vaccination series was chosen (Cohort 10). The sponsor provided 5 randomly selected individuals from this cohort across collected timepoints to balance sample availability with expected variability from previous serological reporting (Widge et al 2021 NEJM). For DMID 22-0004, sample selection was limited to individuals with observed infection independent of enrollment arm. A larger population, limited by sample availability and testing throughput, of N=10 from BA.5 and N=10 from XBB.1.5 infections were randomly selected as vaccination arm and time from infection/vaccination could not be ontrolled for.
Data exclusions	No data were excluded.
Replication	Samples were assayed in duplicate titrations on a plate. Each plate contained a virus-and cell-only (no sample) and cell-only (no virus or sample) controls. Each test run included a positive control for tracking.
Randomization	Randomization for sample analysis was not performed. Randomization strategies during the conduct of the clinical trials are described in detail in the respective clinical protocols.
Blinding	No blinding was performed as the serological analyses performed in this study did not require blinding to maintain rigor.

Reporting for specific materials, systems and methods

We require information from authors about some types of materials, experimental systems and methods used in many studies. Here, indicate whether each material, system or method listed is relevant to your study. If you are not sure if a list item applies to your research, read the appropriate section before selecting a response.

Materials & experimental systems

n/a	Involved in the study
☒	☐ Antibodies
☐	☒ Eukaryotic cell lines
☒	☐ Palaeontology and archaeology
☒	☐ Animals and other organisms
☐	☒ Clinical data
☒	☐ Dual use research of concern
☒	☐ Plants

Methods

n/a	Involved in the study
☒	☐ ChIP-seq
☒	☐ Flow cytometry
☒	☐ MRI-based neuroimaging

Eukaryotic cell lines

Policy information about cell lines and Sex and Gender in Research

Cell line source(s)	293T-human ACE2 cells (obtained from Drs. Michael Farzan and Huihui Mu at UF Scripps Institute) were used for pseudovirus neutralization assays. Related cell lines and pseudovirus propogation all derive from transfection of HEK-293 cells, which were produced when an embryonic kidney cell culture (female).
Authentication	HEK-293 cells were not authenticated.
Mycoplasma contamination	The cell lines used for pseudovirus neutralization were tested monthly and found to be negative for mycoplasma contamination.
Commonly misidentified lines (See ICLAC register)	No commonly misidentified lines were used in this study.

Clinical data

Policy information about clinical studies
All manuscripts should comply with the ICMJE guidelines for publication of clinical research and a completed CONSORT checklist must be included with all submissions.

Clinical trial registration	NCT04283461; NCT05289037
Study protocol	Study protocols are included in the appendices of Jackson et al NEJM 2020 and Branche et al Nat Med 2023, respectively
Data collection	Trial data collection information is included in detail in Jackson et al NEJM 2020 and Branche et al Nat Med 2023. Infection and collection dates for DMID 22-0004 are listed in Supplemental Table 2.
Outcomes	A list of outcomes are included in Jackson et al NEJM 2020 and Branche et al Nat Med 2023. Analyses reported here were performed as secondary research with Sponsor approval.

Plants

Seed stocks	N/A
Novel plant genotypes	N/A
Authentication	N/A
