# Supplementary material for: SARS-CoV-2 vaccination and infection elicit cross-neutralizing responses against clade 3 and 4 sarbecoviruses
Source: Nat Commun. 2026 Apr 16;17:5245. doi: 10.1038/s41467-026-71662-y (PMC13260318; doi:10.1038/s41467-026-71662-y)
Supplement: Supplementary file 5 — Transparent Peer Review file [file 41467_2026_71662_MOESM5_ESM.pdf]

nature portfolio

Peer Review File

SARS-CoV-2 Vaccination and Infection Elicit Cross-Neutralizing Responses against Clade 3 and 4 Sarbecoviruses

Corresponding Author: Dr Leonid Serebryannyy

This file contains all reviewer reports in order by version, followed by all author rebuttals in order by version.

Version 0:

Reviewer comments:

Reviewer #1

(Remarks to the Author)
In this study Schmidt and co-authors describe the cross-neutralizing activity of pseudotyped lentiviruses expressing spike proteins of sarbecoviruses clade 3 and 4 by the sera of individuals that received the primary series of COVID-19 mRNA vaccines and convalescent individuals infected with BA.5 or XBB.1.5 variants. The authors report that the magnitude of responses in both cohorts against clade 3 and clade 4 sarbecoviruses is comparable to titers observed against SARS-CoV-2 variants. Schmidt and colleagues conclude that these findings suggest the current immunologic landscape, shaped by vaccination and infection, may confer some level of immunity against a variety of clade 3 and clade 4 sarbecoviruses, which could inform future pandemic preparedness and pan-sarbecovirus countermeasure development.
The study is interesting in scope and relevant considering the constant pandemic threat posed by sarbecoviruses. However, previous studies have already reported that SARS-CoV-2 vaccination and/or infection can elicit antibodies capable of cross-neutralizing sarbecoviruses beyond clade 1b, including clade 1a, 1c (alias clade 4), and certain clade 3 viruses. For example, Feng et al. 2023 (Sci. Transl. Med.) demonstrated that macaques immunized with an AS03-adjuvanted SARS-CoV-2 subunit vaccine produced monoclonal antibodies capable of neutralizing clade 3 sarbecoviruses such as BtKY72 and PRD-0038. Hu et al. 2024 (NPJ Vaccines), reported that sera from vaccinated and/or convalescent humans contain cross-neutralizing antibodies against a panel of zoonotic sarbecoviruses spanning multiple clades, including clade 4 (1c). These prior findings indicate that the novelty of the present work is somewhat limited.
In addition, some results raise questions about the strength of the conclusions. Specifically, the cross-neutralization of sera from SARS-CoV-2 vaccinated or BA.5/XBB.1.5 convalescent individuals against lentiviral particles representing clade 3 is not paralleled by a significant neutralization against clade 1a (SARS-CoV). This pattern is unexpected given that the receptor-binding domain (RBD) of SARS-CoV-2 and SARS-CoV-2 variants is phylogenetically closer to SARS-CoV (clade 1a) than to clade 3, raising questions about the underlying immunological mechanisms.

Major points:
Considering the number of publications that classify the sarbecoviruses representative of “clade 4” in this work as “clade 1c” (e.g. Si et al. 2024, Nat. Commun.; Lee et al. 2023 Nat Commun, Kosugi et al. 2025 Cell Reports) it would be beneficial to have this interchangeability stated upfront in the main text.

Related to Figure 1:
•It is interesting that sera from individuals vaccinated with ancestral SARS-CoV-2 were able to neutralize lentiviral particles representative of clade 4 (1c) and 3 but showed no neutralization against clade 1a (SARS-CoV). Considering that the RBD of SARS-CoV-2 and Omicron variants is closer to SARS-CoV than to clade 3 viruses, the authors should clarify why there is no neutralization against SARS-CoV S-based lentiviral particles.
•Symbols in the figure are too small, and the colours for Day 29 and Day 57 are too similar. Consider using distinct symbols rather than relying solely on colour differences.

Related to Figure 2:
•Panel 2A is visually quite dense, making it difficult for the reader to follow the fold-changes in neutralizing activity by BA.5 and XBB.1.5 convalescent patients. Please consider dividing the panel into two separate subpanels (BA.5 and XBB.1.5) for clarity.

•Similar to vaccinated individuals, sera from BA.5 or XBB.1.5 convalescent patients showed higher neutralizing activity against clades 4 (1c) and 3 than clade 1a (SARS-CoV). The authors should discuss possible immunological explanations for this unexpected pattern.
•Symbols are too small; Please consider using different symbols rather than different colors.

Related to Figure 3:
•The paragraph describing sera neutralization against wild-type clade 3 and 4 (1c) pseudoviruses appears limited in scope. It would be interesting for instance to have an indication of the entry of the lentiviral particles in cells expressing huACE2, R. affinis 9479 ACE2, R. alcyone ACE2, P. abramus ACE2, and P. sordida.
•A statistical comparison of cross-neutralizing activity between sera from DMID 20-0003 and DMID 22-004 participants would help determine whether observed differences are significant.

References
Feng, S., et al. Broadly neutralizing antibodies against sarbecoviruses generated by immunization with an AS03-adjuvanted SARS-CoV-2 subunit vaccine in macaques. Sci. Transl. Med. 15, eabq4416 (2023).
Hu, B., et al. Broad cross-neutralizing antibodies against sarbecoviruses generated by SARS-CoV-2 infection and vaccination in humans. NPJ Vaccines 9, 112 (2024).
Si, J. Y., et al. Sarbecovirus RBD indels and specific residues dictating multi-species ACE2 adaptiveness. Nat Commun 15, 8869 (2024). https://doi.org/10.1038/s41467-024-53029-3
Lee, J., et al. Broad receptor tropism and immunogenicity of a clade 3 spike. Nat Commun 14, 9201 (2023).
Kosugi, Y., et al. Evolutionary and structural insights into bat and pangolin sarbecoviruses. Cell Reports 41, 112500 (202

Reviewer #2

(Remarks to the Author)
This manuscript comprehensively defines the neutralization landscape against clade 3 and clade 4 animal sarbecoviruses following COVID-19 mRNA vaccination and subsequent infection with BA.5 or XBB.1.5. The study provides valuable insight into how pre-existing SARS-CoV-2 immunity (from vaccination and/or infection) might restrict zoonotic spillover of related sarbecoviruses into humans. The data indicate that two doses of COVID-19 mRNA vaccine elicit measurable neutralizing activity against most clade 3 and clade 4 sarbecoviruses, and that both breadth and potency are enhanced after breakthrough infection, particularly following XBB.1.5 infection in vaccinated cohorts. These findings suggest that the current human population may possess partial protective immunity against clade 3 and clade 4 animal sarbecoviruses, potentially establishing an immunological barrier to spillover.
Major Comments
1. Sequence and Antigenic Relationships
It would strengthen the manuscript to include a table or matrix comparing spike or RBD sequence identities of the clade 3 and clade 4 viruses used in neutralization assays to the ancestral WA1 (or D614G) SARS-CoV-2 strain, as well as to BA.5 and XBB.1.5. Such comparisons could clarify the antigenic distance hierarchy observed in the antigenic map. A phylogenetic tree depicting these relationships would further aid interpretation.
2. Binding Antibody Measurements
Since neutralization is not the sole correlate of protection (Goldblatt et al., Immunol Rev 2022; Corbett et al., Science 2021; Gilbert et al., Science 2022), including ELISA-based measurements of binding antibodies (to both RBD and spike) would be important to evaluate the level of protection from non-neutralizing antibodies.
3. Inclusion of Additional Strains
For completeness, the authors could consider testing additional sarbecoviruses known to exhibit reduced neutralization sensitivity to serum from COVID-19 vaccination/ omicron breakthrough infection, such as Khosta-2 (Seifert et al., PLoS Pathog 2022). It would also be useful to include other clade 1a viruses like SHC014 (Mencachery et al., Nature Medicine 2015). These viruses have been shown to pose a potential risk for human emergence.
Minor Comments
• The legends for Figures 1b and 2b appear blurry and should be improved for readability.
• In Figure 1, the label for panel B should be positioned slightly lower for consistency.

Reviewer #3

(Remarks to the Author)
This is an interesting paper that examines the breadth of human serum NAb activity against diverse sarbecoviruses, including clade 3 + 4 viruses. The authors utilize a panel of pseudotyped lentiviruses expressing the Spikes from representative sarbecoviruses to profile NAb responses in individuals who received COVID-19 mRNA vaccines, as well as in those infected with SARS-2 variants.

The study is well designed and addresses an important point in our understanding of the immunologic landscape to SARS-related CoVs. The finding that vaccination and breakthrough infection elicit neut responses against clade 3 and 4 sarbecoviruses is important. Particularly important is the observation that infection with a variant boosts neut titers across a wide range of sarbecoviruses, suggesting that recent variant exposures may broaden cross-sarbecovirus immunity.

Overall, the manuscript is well executed and clearly written. It provides valuable insights into the cross-neutralizing potential of vaccine and infection induced Ab responses and has important implications for future pandemic preparedness and the

development of pan-sarbecovirus vaccines and countermeasures. My only minor suggestion is to include prior literature showing that CoV infection/vaccination induces cross-reactive Ab responses, as suggested below:

Minor:

The authors should cite previous work showing that SARS-2 infection or vaccination elicit cross-reactive immune responses.
PMID: 33500101
PMID: 41123344
PMID: 34623973

Version 1:

Reviewer comments:

Reviewer #1

(Remarks to the Author)
Serebryannyy and co-authors have addressed the reviewer’s comments.
In particular, the authors have incorporated appropriate references to previously published work on the same topic, which helps to better contextualize the present study within existing literature. The expanded discussion now more clearly delineates how the current study builds upon and differs from prior reports, thereby providing a stronger understanding of its specific contributions and strengths.

Furthermore, the authors acknowledged that conservation of specific neutralizing epitopes is not necessarily reflected in cross-clade phylogeny comparisons, reinforcing the need to better characterize these epitopes in future studies.

Finally, the revised graphs are now presented with greater clarity and are much easier to interpret, which will significantly increase the reader’s ability to follow and understand the results.

Reviewer #2

(Remarks to the Author)
I thank the authors for their thorough revisions. All of my concerns have been addressed, and I am satisfied with the revised manuscript.

Open Access This Peer Review File is licensed under a Creative Commons Attribution 4.0 International License, which permits use, sharing, adaptation, distribution and reproduction in any medium or format, as long as you give appropriate credit to the original author(s) and the source, provide a link to the Creative Commons license, and indicate if changes were made.

In cases where reviewers are anonymous, credit should be given to 'Anonymous Referee' and the source.
The images or other third party material in this Peer Review File are included in the article's Creative Commons license, unless indicated otherwise in a credit line to the material. If material is not included in the article's Creative Commons license and your intended use is not permitted by statutory regulation or exceeds the permitted use, you will need to obtain permission directly from the copyright holder.
To view a copy of this license, visit https://creativecommons.org/licenses/by/4.0/

Responses to reviewer comments:

We greatly appreciate the time and effort spent reviewing this study, and the insightful comments raised. These have helped us to improve the quality of this manuscript. Below, we provide detailed point-by-point responses.

Reviewer #1 (Remarks to the Author):

In this study Schmidt and co-authors describe the cross-neutralizing activity of pseudotyped lentiviruses expressing spike proteins of sarbecoviruses clade 3 and 4 by the sera of individuals that received the primary series of COVID-19 mRNA vaccines and convalescent individuals infected with BA.5 or XBB.1.5 variants. The authors report that the magnitude of responses in both cohorts against clade 3 and clade 4 sarbecoviruses is comparable to titers observed against SARS-CoV-2 variants. Schmidt and colleagues conclude that these findings suggest the current immunologic landscape, shaped by vaccination and infection, may confer some level of immunity against a variety of clade 3 and clade 4 sarbecoviruses, which could inform future pandemic preparedness and pan-sarbecovirus countermeasure development.
The study is interesting in scope and relevant considering the constant pandemic threat posed by sarbecoviruses. However, previous studies have already reported that SARS-CoV-2 vaccination and/or infection can elicit antibodies capable of cross-neutralizing sarbecoviruses beyond clade 1b, including clade 1a, 1c (alias clade 4), and certain clade 3 viruses. For example, Feng et al. 2023 (Sci. Transl. Med.) demonstrated that macaques immunized with an AS03-adjuvanted SARS-CoV-2 subunit vaccine produced monoclonal antibodies capable of neutralizing clade 3 sarbecoviruses such as BtKY72 and PRD-0038. Hu et al. 2024 (NPJ Vaccines), reported that sera from vaccinated and/or convalescent humans contain cross-neutralizing antibodies against a panel of zoonotic sarbecoviruses spanning multiple clades, including clade 4 (1c). These prior findings indicate that the novelty of the present work is somewhat limited.
In addition, some results raise questions about the strength of the conclusions. Specifically, the cross-neutralization of sera from SARS-CoV-2 vaccinated or BA.5/XBB.1.5 convalescent individuals against lentiviral particles representing clade 3 is not paralleled by a significant neutralization against clade 1a (SARS-CoV). This pattern is unexpected given that the receptor-binding domain (RBD) of SARS-CoV-2 and SARS-CoV-2 variants is phylogenetically closer to SARS-CoV (clade 1a) than to clade 3, raising questions about the underlying immunological mechanisms.
Response: We thank the reviewer for their insights and recognition for the potential implications of this work in pandemic preparedness. As the reviewer notes, previous work has implied that SARS-CoV-2 responses may extend to other clades. We now reference the contributions by Hu et

al and Feng et al in the discussion and emphasize how our results build on these previous observations with a broader panel and more contemporaneous sample sets (lines 165 to 172).

We have added a more in-depth discussion of the difference in phylogenetic and antigenic distances between clades (lines 150 to 164 and Supplemental Figure 3). While CoV-1 may be closer phylogenetically than the clade 3 sarbecoviruses, we find that the homology differs rather similarly between clade 1a to clade 1b as it does between clade 1a to clade 3. This suggests that we need to define the epitopes for these conserved neutralization responses rather than comparing bulk homology. While identifying these epitopes is out of scope of the current study, we are beginning follow-up studies to profile cross-reactive B-cells in these cohorts.

Major points:

Considering the number of publications that classify the sarbecoviruses representative of "clade 4" in this work as "clade 1c" (e.g. Si et al. 2024, Nat. Commun.; Lee et al. 2023 Nat Commun, Kosugi et al. 2025 Cell Reports) it would be beneficial to have this interchangeability stated upfront in the main text.

Response: We thank the reviewer for pointing this out and now note that clade 4 is also known as clade 1c in the abstract, on line 48, and have annotated this in the figures as well.

Related to Figure 1:

•It is interesting that sera from individuals vaccinated with ancestral SARS-CoV-2 were able to neutralize lentiviral particles representative of clade 4 (1c) and 3 but showed no neutralization against clade 1a (SARS-CoV). Considering that the RBD of SARS-CoV-2 and Omicron variants is closer to SARS-CoV than to clade 3 viruses, the authors should clarify why there is no neutralization against SARS-CoV S-based lentiviral particles.

•Symbols in the figure are too small, and the colours for Day 29 and Day 57 are too similar. Consider using distinct symbols rather than relying solely on colour differences.

Response: As the reviewer suggests, we have adjusted the color differences between days and now use distinct symbols.

Related to Figure 2:

•Panel 2A is visually quite dense, making it difficult for the reader to follow the fold-changes in neutralizing activity by BA.5 and XBB.1.5 convalescent patients. Please consider dividing the panel into two separate subpanels (BA.5 and XBB.1.5) for clarity.

•Similar to vaccinated individuals, sera from BA.5 or XBB.1.5 convalescent patients showed higher neutralizing activity against clades 4 (1c) and 3 than clade 1a (SARS-CoV). The authors should discuss possible immunological explanations for this unexpected pattern.

•Symbols are too small; Please consider using different symbols rather than different colors.

Response: As the reviewer suggests, we have split the BA.5 and XBB.1.5 donor graphs and now use different symbols for each group.

Related to Figure 3:

•The paragraph describing sera neutralization against wild-type clade 3 and 4 (1c) pseudoviruses appears limited in scope. It would be interesting for instance to have an indication of the entry of the lentiviral particles in cells expressing huACE2, R. affinis 9479 ACE2, R. alcyone ACE2, P. abramus ACE2, and P. sordida.

Response: The signal generated with entry from all pseudoviruses used in the study are listed in Supplemental Table 1. This supplemental table has been renamed to be more explicit.

•A statistical comparison of cross-neutralizing activity between sera from DMID 20-0003 and DMID 22-004 participants would help determine whether observed differences are significant.

Response: There are no significant differences between DMID 20-0003 Day 57 and the pre-infection timepoint for DMID 22-0004. Supplemental Figure 2 has been added to better show this comparison. Line 104 has been changed to note that the activity is comparable between these cohorts/timepoints.

References

Feng, S., et al. Broadly neutralizing antibodies against sarbecoviruses generated by immunization with an AS03-adjuvanted SARS-CoV-2 subunit vaccine in macaques. Sci. Transl. Med. 15, eabq4416 (2023).

Hu, B., et al. Broad cross-neutralizing antibodies against sarbecoviruses generated by SARS-CoV-2 infection and vaccination in humans. NPJ Vaccines 9, 112 (2024).

Si, J. Y., et al. Sarbecovirus RBD indels and specific residues dictating multi-species ACE2 adaptiveness. Nat Commun 15, 8869 (2024). https://doi.org/10.1038/s41467-024-53029-3

Lee, J., et al. Broad receptor tropism and immunogenicity of a clade 3 spike. Nat Commun 14, 9201 (2023).

Kosugi, Y., et al. Evolutionary and structural insights into bat and pangolin sarbecoviruses. Cell Reports 41, 112500 (202

Reviewer #2 (Remarks to the Author):

This manuscript comprehensively defines the neutralization landscape against clade 3 and clade 4 animal sarbecoviruses following COVID-19 mRNA vaccination and subsequent infection with BA.5 or XBB.1.5. The study provides valuable insight into how pre-existing SARS-CoV-2 immunity

(from vaccination and/or infection) might restrict zoonotic spillover of related sarbecoviruses into humans. The data indicate that two doses of COVID-19 mRNA vaccine elicit measurable neutralizing activity against most clade 3 and clade 4 sarbecoviruses, and that both breadth and potency are enhanced after breakthrough infection, particularly following XBB.1.5 infection in vaccinated cohorts. These findings suggest that the current human population may possess partial protective immunity against clade 3 and clade 4 animal sarbecoviruses, potentially establishing an immunological barrier to spillover.

Major Comments

1. Sequence and Antigenic Relationships

It would strengthen the manuscript to include a table or matrix comparing spike or RBD sequence identities of the clade 3 and clade 4 viruses used in neutralization assays to the ancestral WA1 (or D614G) SARS-CoV-2 strain, as well as to BA.5 and XBB.1.5. Such comparisons could clarify the antigenic distance hierarchy observed in the antigenic map. A phylogenetic tree depicting these relationships would further aid interpretation.

Response: We thank the reviewer for their encouraging comments. We now include a phylogeny tree and homology matrix for the RBDs of the pseudoviruses used in Supplemental Figure 3. We also discuss that phylogeny for inter-clade comparisons are not as informative as may have been the case for CoV-2 lineage mutations on lines 150 to 164.

2. Binding Antibody Measurements

Since neutralization is not the sole correlate of protection (Goldblatt et al., Immunol Rev 2022; Corbett et al., Science 2021; Gilbert et al., Science 2022), including ELISA-based measurements of binding antibodies (to both RBD and spike) would be important to evaluate the level of protection from non-neutralizing antibodies.

Response: While binding and neutralization responses generally correlate, we wholly agree with the reviewer that binding antibodies (and associated Fc-mediated functions) and T-cell responses have a role in disease protection as the reviewer has noted and has been implied from our previous studies (Corbett et al., Science 2021, Gilbert et al., Science 2022). However, neutralization titer is likely still the best correlate for risk and protection (Fong et al, Nature Microbiology, 2022; Carpp et al, Nature Communications 2024; Benkeser, Science Translational Medicine 2023). We now highlight our focus on only neutralization titers as a limitation of the study on lines 181 to 185.

To the reviewer’s point, we are planning a future study that compares binding with neutralization responses in non-human primates vaccinated with spike proteins across clades 1 to 4 to better understand the breadth that can be conferred.

3. Inclusion of Additional Strains

For completeness, the authors could consider testing additional sarbecoviruses known to exhibit reduced neutralization sensitivity to serum from COVID-19 vaccination/ omicron breakthrough infection, such as Khosta-2 (Seifert et al., PLoS Pathog 2022). It would also be

useful to include other clade 1a viruses like SHC014 (Mencachery et al., Nature Medicine 2015). These viruses have been shown to pose a potential risk for human emergence.

Response: As the reviewer suggests, we now include neutralization data and mapping for SHC014 and Khosta-2.

Minor Comments
• The legends for Figures 1b and 2b appear blurry and should be improved for readability.
• In Figure 1, the label for panel B should be positioned slightly lower for consistency.

Response: As the reviewer suggests, we have remade the figure legend to be less blurry and have adjusted the labeling.

Reviewer #3 (Remarks to the Author):

This is an interesting paper that examines the breadth of human serum NAb activity against diverse sarbecoviruses, including clade 3 + 4 viruses. The authors utilize a panel of pseudotyped lentiviruses expressing the Spikes from representative sarbecoviruses to profile NAb responses in individuals who received COVID-19 mRNA vaccines, as well as in those infected with SARS-2 variants.

The study is well designed and addresses an important point in our understanding of the immunologic landscape to SARS-related CoVs. The finding that vaccination and breakthrough infection elicit neut responses against clade 3 and 4 sarbecoviruses is important. Particularly important is the observation that infection with a variant boosts neut titers across a wide range of sarbecoviruses, suggesting that recent variant exposures may broaden cross-sarbecovirus immunity.

Overall, the manuscript is well executed and clearly written. It provides valuable insights into the cross-neutralizing potential of vaccine and infection induced Ab responses and has important implications for future pandemic preparedness and the development of pan-sarbecovirus vaccines and countermeasures. My only minor suggestion is to include prior literature showing that CoV infection/vaccination induces cross-reactive Ab responses, as suggested below:

Minor:

The authors should cite previous work showing that SARS-2 infection or vaccination elicit cross-reactive immune responses.

PMID: 33500101

PMID: 41123344

PMID: 34623973

Response: We thank the reviewer for their positive feedback. We have included PMID: 34623973 and PMID: 33500101 in our discussion of clade 1a cross-reactive responses on lines 168 to 170.
